# Differences between two populations of bollworm, *Helicoverpa zea* (Lepidoptera: Noctuidae), with variable measurements of laboratory susceptibilities to Bt toxins exposed to non-Bt and Bt cottons in large field cages

**DOI:** 10.1371/journal.pone.0212567

**Published:** 2019-03-13

**Authors:** Nathan S. Little, Blake H. Elkins, R. Michelle Mullen, Omaththage P. Perera, Katherine A. Parys, K. Clint Allen, Deborah L. Boykin

**Affiliations:** 1 Southern Insect Management Research Unit, USDA-ARS, Stoneville, Mississippi, United States of America; 2 Jamie Whitten Delta States Research Center, USDA-ARS, Stoneville, Mississippi, United States of America; Nigde Omer Halisdemir University, TURKEY

## Abstract

Interpreting variable laboratory measurements of *Helicoverpa zea* Boddie susceptibility to toxins from *Bacillus thuringiensis* Berliner (*Bt*) has been challenging due to a lack of clear evidence to document declining field control. Research that links laboratory measurements of susceptibility to survival on *Bt* crops is vital for accurate characterization and any subsequent response to the occurrence of an implied *H*. *zea* resistance event. In this study, *H*. *zea* survival and the resultant damage to plant fruiting structures of non-*Bt*, Bollgard II, and Bollgard III cottons from two insect colonies with differing levels of laboratory susceptibility to *Bt* toxins were evaluated in large field cages. Laboratory bioassays revealed resistance ratios of 2.04 and 622.14 between the two *H*. *zea* colonies for Dipel DF and Cry1Ac, respectively. Differences between the two *H*. *zea* colonies measured via bioassays with Bollgard II and Bollgard III cotton leaf tissue in the laboratory were not statistically discernable. However, there was 17.6% and 5.3% lower larval mortality in Bollgard II and Bollgard III for the feral relative to the laboratory colony of *H*. *zea*, respectively. Although *H*. *zea* larval numbers in cages infested with the laboratory susceptible colony did not differ between the two *Bt* cottons, there were fewer larvae per 25 plants in Bollgard III than in Bollgard II cotton in cages containing tolerant insects. Cages infested with tolerant *H*. *zea* moths had higher numbers of total larvae than those containing the laboratory susceptible colony in both Bollgard II and Bollgard III cottons. Bollgard II and Bollgard III cottons received 77.4% and 82.7% more total damage to total plant fruiting structures in cages infested with tolerant insects relative to those containing the laboratory susceptible colony. The damage inflicted to fruiting structures on Bollgard III cotton by a feral *H*. *zea* colony with decreased measurements of laboratory susceptibility to Dipel DF and Cry1Ac indicate that the addition of Vip3A to third generation *Bt* cottons may not provide sufficient control in situations where infestations levels are high.

## Introduction

Following the introduction and subsequent widespread adoption of genetically modified crops expressing crystalline (Cry) protein(s) produced by *Bacillus thuringiensis* Berliner (*Bt*) to control heliothines in the United States, *Helicoverpa zea* Boddie remains a major pest of cotton. The primary pest targeted for control with *Bt* cotton (*Gossypium hirsutum* L.) in the United States was *Chloridea virescens* (F.); however, this technology has also provided suppression of *H*. *zea* [1]. Nevertheless, producers and pest managers in the U.S. rely heavily on Bt toxins for the control of *H*. *zea* in cotton. Laboratory bioassays have been the primary mechanism used to establish, estimate, and track *H*. *zea* susceptibility to *Bt* toxins [2]. Current assay methods are largely derived from methods used by Dulmage et al. [3], which were developed more than two decades prior to the release of *Bt* crops. Interpreting variable quantitative measurements of *H*. *zea* susceptibility to *Bt* toxins obtained from laboratory bioassays has been challenging due to the lack of clear evidence to document declining field control [2]. Conversely, broad characterizations of field-evolved *H*. *zea* resistance to *Bt* toxins have been based largely on bioassay results (see [4, 5, 6]). Research that links variable measurements of laboratory susceptibility to survival on *Bt* crops is vital for accurate characterization of and any subsequent response to the occurrence of an implied *H*. *zea* resistance event. Unfortunately, the recent introduction of cotton varieties that express multiple *Bt* toxins may further our reliance on laboratory assays for characterization of susceptibility in *H*. *zea* due to a lack of empirical field-based assessments of these new technologies regarding insect survival and damage to plant fruiting structures.

*H*. *zea* is a polyphagous lepidopteran pest that feeds on numerous wild and cultivated crops. Two of its major crop hosts in the southern U.S. are corn (*Zea mays* L.) and cotton (*G*. *hirsutum*). A large proportion of the cropland in the southeastern U.S. is planted with these two crops; the majority of which express two or more *Bt* proteins. A clear majority of *H*. *zea* utilize corn as a second-generation host [7, 8]. This is concerning due to the exposure of multiple successive generations of *H*. *zea* to *Bt* toxins, which have similar modes of insecticidal action, and are found in both corn and cotton cultivars. Due to the season-long expression of *Bt* toxins in plants and the importance of sprayable formulations as a safe insecticide used in organic and vegetable crop production, proactive resistance management plans were implemented with the introduction of *Bt* crops [9]. A combination of “high-dose” and mandated refuge strategies were implemented for the targeted insects of *Bt* crops upon commercial release [9]. Conceptually, this multifaceted strategy assumed that the expression levels in tissues were high enough to kill all but the rare homozygous resistant insects, which would then mate with the heterozygotes that developed on non-*Bt* crops and wild hosts. Although this approach was sufficient for delaying resistance in *C*. *virescens*, the plan was inadequate for *H*. *zea* due to its innate capacity to survive on both *Bt* corn and cotton.

The evolution of *H*. *zea* resistance is a major threat to the continued success of *Bt* cotton in the U.S. [10–16]. Reports and annual assessments of insects targeted by transgenic insecticides were conducted to detect potential shifts in susceptibility to the ever-growing myriad of proteins expressed in *Bt* crops [2]. These assessments consisted of feral populations assayed on both meridic diet incorporated and overlaid with *Bt* toxins and subsequent probit analyses to examine lethal concentration (LC) values relative to susceptible laboratory populations. Results from these assessments were highly variable, and *H*. *zea* was shown to possess an inherent proclivity for tolerance that predated the commercial release of *Bt* crops [17–19]. However, the ultimate ability of these populations to survive on *Bt* plants in the field was generally not known. Although populations of heliothines have been selected for resistance to Cry1Ac in the laboratory [19–21], *C*. *virescens* was reportedly unable to survive on non-*Bt* or *Bt* cotton tissues [12]. Reisig et al. [22] offered the first tangible evidence of field-evolved resistance in *H*. *zea* by linking results from laboratory bioassays with Cry1Ac to seasonal measurements of damaged bolls in pyramided-gene *Bt* cottons. However, a foliar organophosphate (acephate) was applied prior to *H*. *zea* oviposition, which virtually eliminated natural enemies, likely inflating damage estimates. Characterizations of resistance based solely on LC ratios of feral populations relative to laboratory susceptible insects have been an inaccurate predictor of the ability of *H*. *zea* to survive on *Bt* plants.

*Bt* expression levels in cotton can differ spatially, temporally, and with changes in environmental conditions [23–28] and are known to decrease substantially following bloom [29, 30]. Differences in expression levels have also been documented within plants by tissue type [31], variety [23, 32], and among plants [25]. Observations of increased survival in the field could be due to decreases in susceptibility to the toxins or other factors such as choosing to feed on tissues with lower expression levels of *Bt* toxins [31]. Documenting insect survival, and the resultant damage on tissue types of *Bt* plants compared to non-*Bt* plants provides additional information regarding potential shifts in susceptibility.

Quantifying the relationship between the relative susceptibility of *H*. *zea* to *Bt* proteins as measured in laboratory bioassays to the actual survival and damage on *Bt* plants would be an important step in understanding the economic impact of insects with varying degrees of susceptibility on cotton. In this study, we initiated experiments to link insect survival and damage on dual- and multi-gene cotton plants with laboratory estimates of two *H*. *zea* colonies with different levels of susceptibility to *Bt* toxins. This information will allow for better predictions of *H*. *zea* control in *Bt* cottons based on laboratory measurements of susceptibility to *Bt* toxins. This will be especially important with the release of new cotton varieties, such as those that express Vip3A.

## Materials and methods

### Laboratory assays

A suite of laboratory assays were conducted to characterize the susceptibility of two separate colonies of *H*. *zea* to *Bt* toxins using a commercially-formulated product (Dipel DF, Valent BioSciences, Libertyville, IL), cotton leaf tissue [non-*Bt* (DP1441RF, Delta and Pine Land Company, Scott, MS), Bollgard II (DP1321B2RF, Delta and Pine Land Company, Scott, MS), and Bollgard III (DP1835B3XF, Delta and Pine Land Company, Scott, MS)], and purified Cry1Ac. Cry1Ac toxin used in the experiments was produced using *B*. *thuringiensis* strain HD-73 obtained from the Bacillus GeneticStock Center (Ohio, USA) with a few modifications to a previously described protocol [33]. Briefly, 500 ml of 1/3 strength tryptic soy broth was inoculated with 500 μl of overnight culture that originated from a single colony of the HD-73 strain. Cultures were grown in a shaker-incubator at 28°C for 3 days at 160 rpm. At the end of incubation, cultures were pelleted by centrifuging at 8000 xg for 35 minutes using a JA-10 rotor (Beckman Coulter, Brea, CA). The pellets were resuspended in 50 ml of wash solution consisting of 1M NaCl with 0.1% Triton X-100 and sonicated on ice for 4.5 minutes in cycles of 30 second pulses with a 5 second pause using a Q-Sonica CL-18 sonicator (Q-Sonica, Newtown, CT). Approximately 150 ml of wash solution was added to the sonicated samples and centrifuged for 10 minutes at 17,000 xg. This process was repeated two additional times and the resulting crystalline inclusions were solubilized by incubation overnight at 30°C in 50 mM Na_2_CO_3_ pH 9.8, 100 mM NaCl 0.1% 2-β-mercaptoethanol. After solubilization, samples were clarified by centrifugation at 30,000 xg for 20 min at 4°C. Cry1Ac protoxin in the supernatant was concentrated using a Centricon Plus-70 centrifugal filter device (Millipore, Burlington, MA) and quantified by electrophoresis on polyacrylamide gels with Cry1Ac and BSA standards.

### Insect colonies

A laboratory-reared *H*. *zea* colony has been consecutively reared for over 500 generations at the United States Department of Agriculture Agricultural Research Service (USDA ARS), Southern Insect Management Research Unit (SIMRU) in Stoneville, MS since 1971. This laboratory colony is routinely reared following details outlined by Blanco et al. [34] for *C*. *virescens* to provide large numbers of insects for assay purposes and is considered susceptible to both synthetic and transgenic insecticides.

A feral *H*. *zea* colony was collected on June 24, 2016 from ears of *Bt* corn (Vt2Pro, Monsanto Company) near Pickens, AR (33.837267, -91.475281). This corn was verified as containing *Bt* using a QuickStix Combo for Cry1A and Cry2A (Catalog #AS-012-LS and #AS-012-LSS, Envirologix, Portland, ME) in the leaf tissue. Larvae (F_0_) were collected from the field and placed in 36.9 ml clear portion containers (Dart Container Corp., Mason, MI) containing meridic diet (Nutri-Soy Wheat Germ) [34]. These insects were returned to the laboratory and reared using methods described by Blanco et al [34]. Pupae were sexed, and upon eclosion, moths were placed in four separate mating arenas at a 50:50 (M:F) ratio. These mating arenas consisted of 3.8 l paper containers, each of which was equipped with a 59 ml plastic cup (Dart Container Corp., Mason, MI) containing cotton balls saturated with a 10% sugar solution for adult *H*. *zea* nourishment. The containers were covered with cheese cloth as an oviposition substrate.

Four replicates of eight doses of Dipel DF (Valent BioSciences, Libertyville, IL) incorporated into meridic diet (0, 3, 10, 30, 100, 300, 1,000, and 3,000 μg/ml) utilizing 16 larvae per replicate and dose, were conducted with neonate larvae (F_1_) from the Pickens, AR colony. These bioassays yielded a 26.2-fold difference (LC_50_ of Pickens, AR colony/LC_50_ of the SIMRU laboratory colony) and a resistance ratio (RR_50_) of 16.05 (10.23–25.2) [35], which was the highest for any field colony tested against Dipel DF during the 2016 cropping season. Survivors (F_1_) from the 1,000 μg/ml dose from all four replicates of the Pickens, AR colony were transferred to untreated meridic diet immediately following the seven-day test. After being maintained for 6 generations (~5 months), these insects were crossed with individuals from the SIMRU lab colony due to declining fecundity and egg viability. Insects from the F_7_ generation were selected on 40 μg Dipel DF/ml of meridic diet (LC_20_ dose from original F_1_ bioassays) prior to being utilized in the field cage study outlined below. Moths from each colony (Pickens, AR and SIMRU lab) were held in respective paper containers in the lab for 24 hours to allow for mating prior to release in the field cage experiment.

#### Field cage design

**Fig 1:**
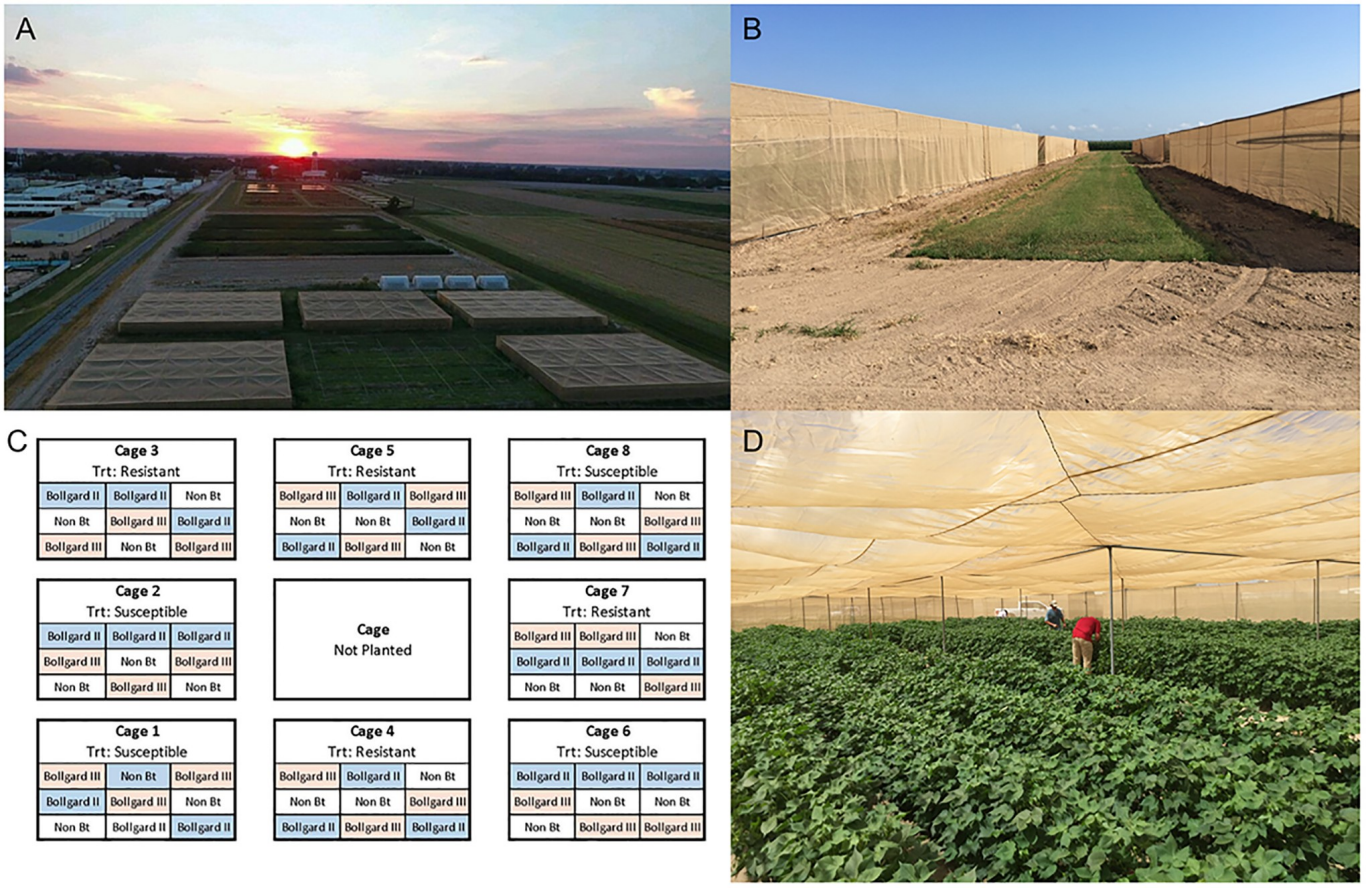
Images showing a) aerial view of cages 1–5, b) interior of a single cage with workers scouting cotton plots, c) experimental cage layout of cotton type and insect treatments, and d) scouting of cotton plots for insect densities and plant damage.

Eight large field cages (30.5 x 18.3 x 2.7 m (l x w x h)) for insect research on the campus of the Jamie Whitten Delta States Research Center have been maintained by USDA-ARS SIMRU near Stoneville, MS since the early 1980s. A single cage contains three sections, each with four rows (96.5 cm wide) running the entire length. Each section within the cages was subdivided into three 9 m units separated by a 1.5 m border on the ends (Fig 1). Each cotton cultivar (non-*Bt*, Bollgard II, and Bollgard III) was randomly assigned and planted in one of the three units for each section, for a total of nine plots per cage. All cotton seed was treated by the manufacturer prior to planting with Acceleron Standard (Monsanto Technology, L.L.C., St. Louis, MO). Cotton was planted on May 10, 2017 with a four-row John Deere 7100 planter (Moline, IL) equipped with ALMACO cone units (Nevada, IA), which were calibrated to deliver 10.5 seeds per meter of row.

Early season non-target insect pests (e.g., tobacco thrips (*Frankliniella fusca* (Hinds)), aphids (Hemiptera: Aphididae), and tarnished plant bugs (*Lygus lineolaris* (Palisot de Beauvois))) were controlled with broadcast sprays of acephate (Bracket 97, Winfield Solutions, LLC, St. Paul, MN) at 1.12 kg/ha, thiamethoxam (Centric 40WG, Syngenta Crop Protection at 0.18 kg/ha, Greensboro, NC), or imidacloprid (Advise 2FL, Winfield Solutions, LLC, St. Paul, MN) at 0.29 liters/ha as needed. During the first week of bloom, broadcast applications of sulfloxaflor (Transform WG, Dow Chemical Company, Indianapolis, IN) and acephate plus bifenthrin (Bifen 2EC, Prime Source, LLC, Middlesex, NC) were rotated to suppress all insect pests prior to installing mesh cage covers. Two separate broadcast applications of mepiquat chloride (Mepiquat, Loveland Products Inc., Greeley, CO) (0.5 l/ha pre-bloom and 1.1 l/ha during the first week of bloom) were applied to manage rapid growth in cotton. Weeds were controlled using ad hoc applications of broadcast and post-direct herbicide applications. All treatment applications were applied in a water solution/suspension with a ground sprayer at 93.5 l/ha. All insecticide treatments were terminated on July 6, 2017. Ammonia sulfate (21-0-0-24) was applied on June 5, 2017 with a tractor spreader at 270 kg/hectare, which supplied 56.7 kg/ha of nitrogen and 64.8 kg/ha of sulfur, and incorporated with a row cultivator.

### Bioassays

A representative subset of the two colonies of *H*. *zea* moths (Pickens, AR and SIMRU lab) used to infest cages were kept in the laboratory and allowed to propagate so that the same generation of insects infesting cotton plots could be simultaneously tested in bioassays. Neonate progeny from both colonies were subjected to five separate assays: 1) meridic diet overlaid with a range of doses of Dipel DF, 2) Cry1Ac incorporated into meridic diet, and leaf tissue assays using 3) non-*Bt*, 4) Bollgard II, and 5) Bollgard III leaves. For diet overlay assays with Dipel DF, repeater pipets were used to dispense 1.5 ml of Nutri-Soy Wheat Germ diet into 128-well bioassay trays (C-D International, Pitman, NJ). After the diet cooled to room temperature, 50 μl of a given concentration of Dipel DF suspended in water was pipetted into each well over the top of the diet. Concentrations of Dipel DF used in diet overlay assays were 0, 0.09, 0.27, 0.82, 2.47, 7.41, 22.22, and 66.67 μg/cm^2^.

Diet incorporated assays were utilized for Cry1Ac. Nutri-Soy Wheat Germ diet was mixed and allowed to cool to 39°C prior to adding Cry1Ac to minimize denaturation. Concentrations of Cry1Ac utilized in assays were 0, 0.03, 0.1, 0.3, 1, 3, 10, and 30 μl/ml diet. The homogenized concentrations were pipetted into the bioassay tray at 0.5 ml/well. Leaf tissue assays with non-*Bt*, Bollgard II, and Bollgard III leaves were conducted as described by Little et al. [36] using 2.54 cm leaf disks in 36.9 ml clear containers with a 5 mm layer of agar. Four replicates were performed for each *H*. *zea* colony, assay type, and one replicate consisted of 16 insects for each concentration and leaf tissue. Neonate larvae (< 1 day old) were used in all assays.

### Cage infestation and data collection

Installation of mesh cage covers began July 10, 2017 and was completed on July 11, 2017. Each of the two *H*. *zea* colonies were randomly assigned to four of the eight cages. Following the 24-h mating period in the laboratory, 300 moths at a 1:1 (M:F) ratio were released in their respective cages between July 17 and July 20, 2017, as they became available. Cotton plots within all cages were scouted July 17, 2017 prior to *H*. *zea* moth releases and weekly beginning July 24, 2017 for number of eggs, number of larvae (≥ 2^nd^ instar) per 25 terminals, blooms, squares, and bolls, and damaged terminals (top five nodes), squares, and bolls per 25 plants. Scouting was terminated on August 18, 2017.

### Analyses

Probit analyses were used to develop regressions for estimating lethal concentrations (LC) of Dipel DF and Cry1Ac needed to kill 50% of neonate *H*. *zea* larvae [37]. Percent mortalities of neonate *H*. *zea* larvae exposed to Bollgard II and Bollgard III leaf disks were corrected for control (non-*Bt*) mortality using Abbott’s formula [38]. Confidence intervals were calculated using Rosenheim and Hoy’s [39] iteration of Elston’s [40] method of correcting assay data for control responses.

Larval abundance and damage to cotton plants were analyzed with a general linear mixed model for a split plot design in SAS 9.4 [37]. The main unit consisted of two insect types (laboratory susceptible and tolerant) with four cages per type arranged in a completely randomized design. The subunit was the three cotton types (non-*Bt*, Bollgard II, and Bollgard III), which had an additional level of replication. There were three blocks within each cage, and the cotton types were randomly assigned within each block. Measurements were taken over time and each rating was analyzed separately using cumulative values, which were calculated so that a measurement at any rating period was the sum of all previous ratings. Each rating period was analyzed using a mixed model with fixed effects for insect type, cotton type, and insect type by cotton type. Random effects included cages within each insect type, blocks within cage, and cage by cotton type within insect type. Many of the cumulative counts were zeros, especially during the early rating periods. The normal distribution assumption required for general linear mixed models was not met. Therefore, a generalized linear mixed model with a negative binomial distribution and a log link function was used to model the data. In cases with an abundance of zeros, some of the random terms were combined into the residual error to facilitate the convergence of models. Pairwise mean comparisons were based on least significant differences (LSDs) at *P* ≤ 0.05 on the log transformed means.

Cotton lint yield was determined by hand harvesting three, 1.52-m row sections in each plot. An estimate of 39% lint yield of seed cotton was used. Yield was analyzed with a general linear mixed model for a split plot design [37]. Least square means were estimated and compared using Fisher’s LSD at α = 0.05 when significant insect type, cotton type, or insect type-by-cotton type effects were detected.

## Results

### Bioassays

Neonate progeny from the Pickens, AR and SIMRU lab colonies subjected to meridic diet overlaid with discriminating doses of Dipel DF yielded LC_50_s of 4.80 and 2.57 μg/cm^2^, respectively. This resulted in a 1.87-fold difference (LC_50_ of Pickens, AR colony/LC_50_ of the SIMRU laboratory colony) and a RR_50_ of 2.04 (1.39–2.99) [35] between the two *H*. *zea* colonies. Bioassays using neonate larvae subjected to Cry1Ac incorporated into meridic diet at the previously given concentrations yielded LC_50_s of 163.62 and 7.76 μl/ml, respectively. These bioassays revealed a 21.08-fold difference (LC_50_ of Pickens, AR colony/LC_50_ of the SIMRU laboratory colony) and a RR_50_ of 622.14 (70.25–5,509.99) [35]. Plant tissue assays, which utilized leaf disks from non-*Bt*, Bollgard II, and Bollgard III cotton plants, resulted in subtle differences in neonate larval mortality. When corrected for non-*Bt* cotton, *H*. *zea* mortalities of the Pickens, AR and SIMRU laboratory colonies on Bollgard II leaf disks were 64.8% (±28.9) and 76.2% (±31.8), respectively. *H*. *zea* mortality on Bollgard III leaf disks was 93.9% (±10.3) and 98.9% (±3.72) for the Pickens, AR and SIMRU laboratory colonies after correction for non-*Bt* tissue.

### Larval densities and plant damage

**Fig 2:**
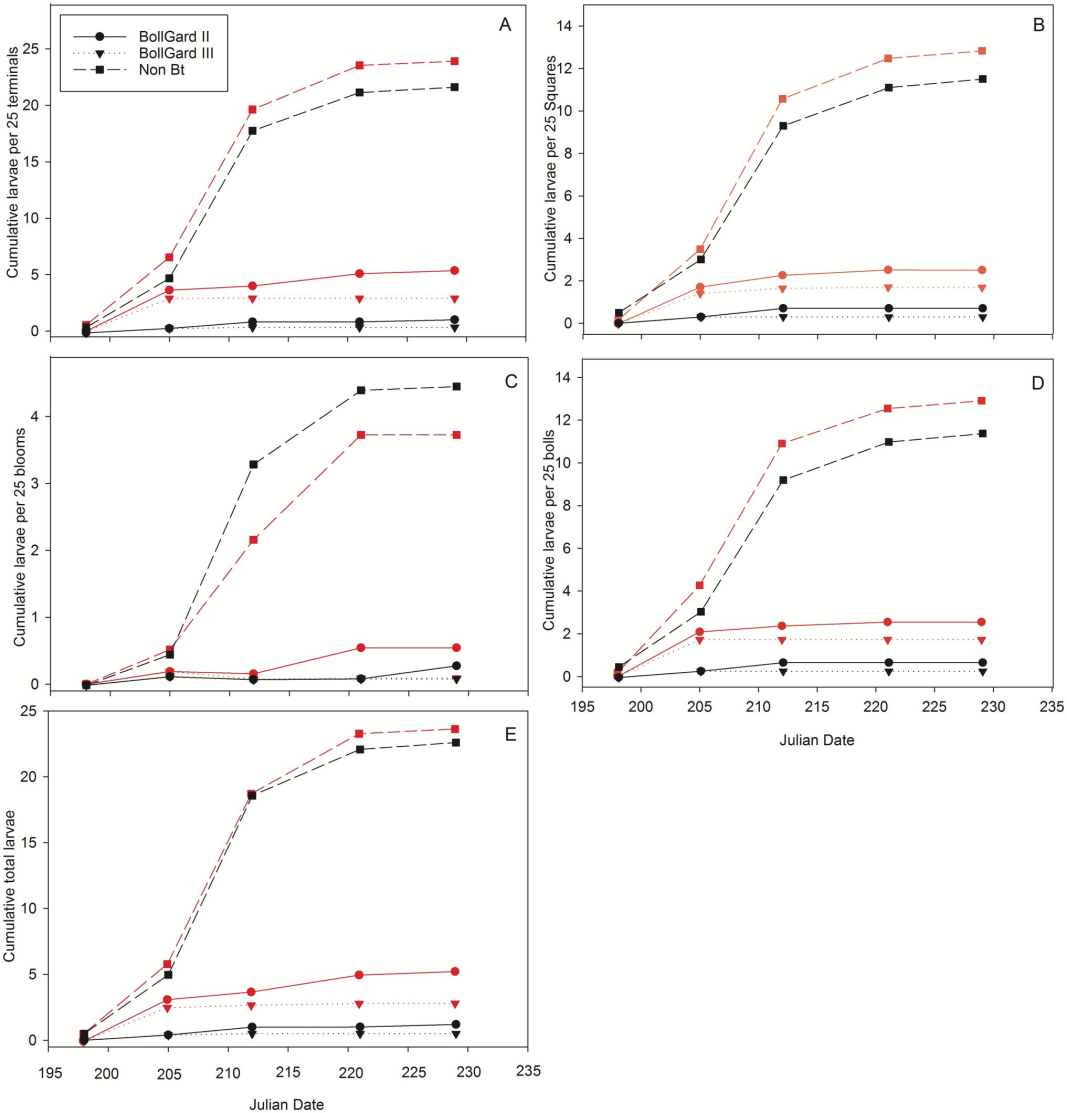
Cumulative larval numbers in cotton a) terminals, b) squares, c) blooms, d) bolls, and e) total larval numbers (sum of terminals, squares, bolls, and blooms) for each cotton type and *H*. *zea* colony over time. Black lines represent the laboratory susceptible *H*. *zea* colony and red lines indicate tolerant insects.

The number of eggs laid by female *H*. *zea* moths among non-*Bt*, Bollgard II, and Bollgard III cottons per 25 plants did not differ regarding the laboratory susceptible and tolerant insect colonies. Moths from the laboratory susceptible colony laid significantly more eggs in Bollgard III per 25 plants than in non-*Bt* cotton (Table 1). *H*. *zea* larval numbers in terminals, squares, bolls, and blooms per 25 plants at the final rating period are shown in Table 2. Cumulative larvae per 25 terminals, squares, bolls, blooms, and total (sum of larvae in terminals, squares, bolls, and blooms) for each cotton type and *H*. *zea* colony at each rating period are displayed in Fig 2. There was no difference in the cumulative number of laboratory susceptible larvae per 25 cotton terminals (top five nodes) among the cotton types. In cages infested with moths from the tolerant colony, non-*Bt* cotton had significantly higher larval numbers per 25 terminals than either of the two *Bt* varieties. There were significantly higher cumulative larval numbers in terminals of Bollgard II cotton in cages infested with tolerant insects than those with the laboratory susceptible colony. Although there were no discernable differences between cumulative larval numbers per 25 squares in the two *Bt* varieties, they both had significantly fewer larvae than non-*Bt* plots. However, there were significantly higher cumulative larval numbers per 25 squares in cages infested with tolerant insects than in those containing the laboratory susceptible colony for both Bollgard II and Bollgard III cottons. Regardless of insect type, there were significantly more cumulative *H*. *zea* larvae per 25 blooms in non-*Bt* cotton than either of the two *Bt* varieties. There were no differences among cotton types regarding cumulative larvae per 25 bolls in cages infested with tolerant insects. However, the two *Bt* varieties had fewer cumulative larvae per 25 bolls than non-*Bt* plots in cages containing the laboratory susceptible colony. Cages infested with tolerant *H*. *zea* moths yielded significantly higher cumulative larval numbers per 25 bolls than those containing laboratory susceptible insects. There were significantly more total cumulative larvae (sum of larvae per 25 terminals, squares, blooms, and bolls) in non-*Bt* cotton than either of the two *Bt* cotton types in cages containing laboratory susceptible insects. However, total cumulative larval numbers of tolerant insects were significantly lower in Bollgard III than in Bollgard II and non-*Bt*. Total larval numbers in Bollgard II cotton were significantly lower than those of non-*Bt* cotton. Furthermore, cumulative total larval numbers per 25 structures were higher in cages infested with tolerant insects than for those containing laboratory susceptible *H*. *zea* for both Bollgard II and Bollgard III cottons.

**Table 1:** Cumulative total *H*. *zea* eggs per 25 plants at final rating period.

Cotton Type	Insect Type	
	Laboratory Susceptible^1^	Tolerant^1^
Non-*Bt*	22.7b	31.2a
Bollgard II	28.6ab	41.3a
Bollgard III	32.3a	42.4a

^1^Means not followed by a common letter indicate significant differences between cotton types within an insect type based on LSD at *P* ≤ 0.05

**Table 2:** Cumulative *H*. *zea* larvae in terminals, squares, bolls, and blooms per 25 plants at final rating period.

Plant Structure	Cotton Type	Insect Type		Significance^2^
		Laboratory Susceptible^1^	Tolerant^1^	
Terminals	Non-*Bt*	2.6a	3.8a	
	Bollgard II	0.1a	1.4b	*
	Bollgard III	0a	1.1b	
Squares	Non-*Bt*	11.3a	12.8a	
	Bollgard II	0.7b	2.5b	*
	Bollgard III	0.3b	1.7b	*
Blooms	Non-*Bt*	4.5a	3.6a	
	Bollgard II	0.3b	0.5b	
	Bollgard III	0.1b	0.1b	
Bolls	Non-*Bt*	3.8a	3.5a	
	Bollgard II	0.1b	0.9a	*
	Bollgard III	0.1b	0a	
Totals^3^	Non-*Bt*	22.6a	23.6a	
	Bollgard II	1.2b	5.3b	*
	Bollgard III	0.5b	2.9c	*

^1^Means not followed by a common letter indicate significant differences between cotton types within an insect type and plant structure based on LSD at *P* ≤ 0.05.

^2^Indicates significant differences between insect type within cotton type. Based on LSD at *P* ≤ 0.05.

^3^Total cumulative larvae across terminals, squares, blooms, and bolls per 25 plants at final rating period.

**Fig 3:**
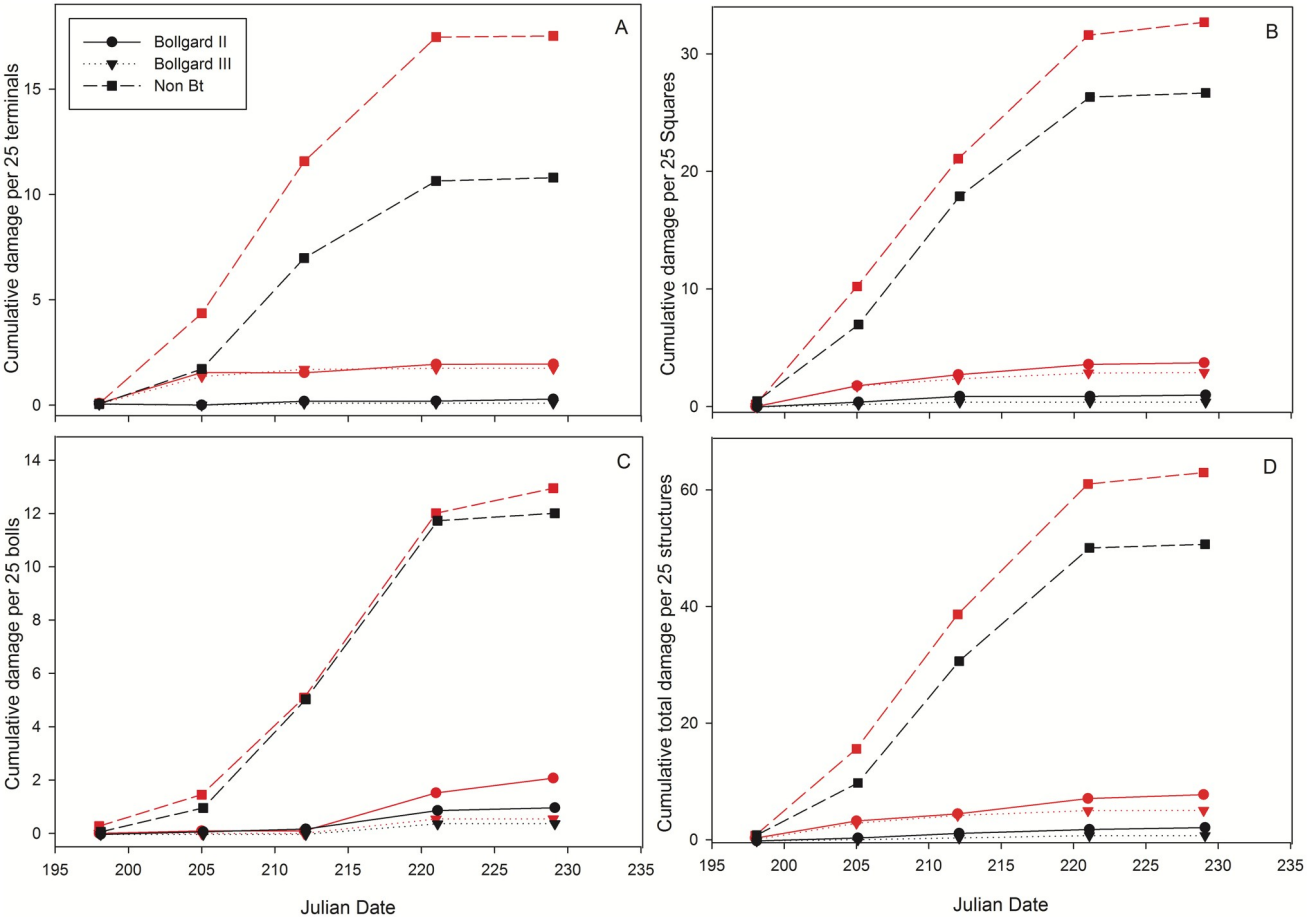
Cumulative damage in cotton a) terminals, b) squares, c) bolls, and d) total plant damage (sum of terminals, squares, and bolls) for each cotton type and *H*. *zea* colony over time. Black lines represent the laboratory susceptible *H*. *zea* colony and red lines indicate tolerant insects.

Damage per 25 terminals, squares, and bolls at the final rating period are shown in Table 3. Cumulative damage per 25 terminals, squares, bolls, and total (sum of damage in terminals, squares, and bolls) for each cotton type and *H*. *zea* colony at each rating period are displayed in Fig 3. Cumulative larval damage per 25 cotton terminals did not differ between two *Bt* cotton varieties within a given insect type. However, both received significantly less damage per 25 cotton terminals than non-*Bt* cotton. Bollgard II and Bollgard III plots in cages infested with tolerant *H*. *zea* moths had significantly higher cumulative terminal damage than those containing laboratory susceptible insects. There was no difference between the two *Bt* cotton types for cumulative damage per 25 squares within a given insect colony, but they both had significantly less damage than those in non-*Bt* cotton. Bollgard II and Bollgard III plots in cages infested with tolerant *H*. *zea* moths had significantly higher cumulative square damage than those containing laboratory susceptible insects. There was no difference between the two *Bt* cotton types for cumulative damage per 25 bolls within cages infested with laboratory susceptible insects, but both had significantly less boll damage than non-*Bt* cotton. In cages infested with tolerant insects, there were significant differences among cotton types regarding cumulative boll damage per 25 structures: Bollgard III < Bollgard II < non-*Bt*. There was no difference between the two *Bt* cotton types regarding total cumulative damage per 25 structures (sum of 25 damaged terminals, squares, and bolls) within a given insect type, but both had significantly less damage than non-*Bt* cotton. Bollgard II and Bollgard III plots in cages infested with tolerant *H*. *zea* moths had significantly higher total cumulative damage than those containing laboratory susceptible insects.

**Table 3:** Cumulative terminal, square, and boll damage caused by *H*. *zea* per 25 plants at final rating period.

Plant Structure	Cotton Type	Insect Type		Significance^2^
		Laboratory Susceptible^1^	Tolerant^1^	
Terminals	Non-*Bt*	11.1a	17.3a	
	Bollgard II	0.3b	1.9b	*
	Bollgard III	0.1b	1.7b	*
Squares	Non-*Bt*	26.6a	32.6a	
	Bollgard II	1b	3.7b	*
	Bollgard III	0.4b	2.9b	*
Bolls	Non-*Bt*	11.9a	12.8a	
	Bollgard II	1b	2.1b	
	Bollgard III	0.4b	0.5c	
Totals^3^	Non-*Bt*	49.6a	61.2a	
	Bollgard II	2.2b	7.6b	*
	Bollgard III	0.9b	4.7b	*

^1^Means not followed by a common letter indicate significant differences between cotton types within an insect type and plant structure based on LSD at *P* ≤ 0.05.

^2^Indicates significant differences between insect type within cotton type. Based on LSD at *P* ≤ 0.05.

^3^Total cumulative damage across terminals, squares, and bolls per 25 structures at final rating period.

### Lint yield

Mean cotton lint yields for each cotton type in cages infested with susceptible and tolerant *H*. *zea* are given in Table 4. There was no significant difference in lint yield between cages infested with tolerant and laboratory susceptible insects among the cotton types. Although lint yields did not differ significantly between the two *Bt* cottons, Bollgard II yielded numerically higher than Bollgard III, which may be due to varietal differences or possibly a cost associated with vegetative production of the Vip3A protein. Both *Bt* varieties had significantly higher lint yields than the non-*Bt* cotton variety.

**Table 4:** Mean lint yields for cotton types exposed to susceptible and tolerant *H*. *zea* in large field cages.

Analysis	Insect type	Cotton Type	Yield^1^ ± SE
Independent^2^	Susceptible	Non-Bt	683.3 ± 78.4b
		Bollgard II	1018.6 ± 78.4a
		Bollgard III	850 ± 78.4ab
	Tolerant	Non-Bt	470.6 ± 75.7b
		Bollgard II	879.6 ± 75.7a
		Bollgard III	868.7 ± 75.7a
Combined^3^	Susceptible	Non-Bt	683.3 ± 95.2c
		Bollgard II	1018.6 ± 95.2a
		Bollgard III	850 ± 95.2ab
	Tolerant	Non-Bt	470.6 ± 93.7c
		Bollgard II	879.6 ± 93.7ab
		Bollgard III	868.7 ± 93.7ab

^1^Mean lint yields are displayed in kg/ha and based on 39% gin-out.

^2^Means within a given insect type and cotton type with the same letter are not significantly based on LSD at *P* ≤ 0.05.

^3^Means with the same letter are not significantly based on LSD at *P* ≤ 0.05.

## Discussion

The capacity for laboratory measurements of *H*. *zea* susceptibility to *Bt* toxins to predict the insect’s ability to survive on transgenic crops in the field remains unclear. Laboratory bioassays are highly controlled while environmental conditions in the field are constantly changing. This can affect key factors associated with insect survival on *Bt* plants such as protein expression and insect behavior and development [24, 41, 42]. At the very least, any implications of insect resistance should be predicated on sound field ecological studies to corroborate decreased measurements of laboratory susceptibility. Therefore, we examined the linkage between laboratory estimates of susceptibility, larval survival, and damage to plant fruiting structures of non-*Bt*, Bollgard II, and Bollgard III cottons in large field cages using two colonies of *H*. *zea* with different levels of susceptibility to *Bt* toxins.

Rearing and maintaining *H*. *zea* colonies that exhibit tolerance to *Bt* proteins presents challenges due to fitness costs associated with insect survival [43–45]. These fitness costs have been reported to be higher when rearing insects on plants compared to those reared on meridic diet in laboratory settings [46]. This is to be expected given the myriad of defensive compounds expressed in plants, either constitutively or in response to herbivory, that affect insect feeding, growth, and survival [47]. Therefore, we would expect a reasonable amount of developmental differences on cotton plants between SIMRU’s laboratory colony of *H*. *zea*, which has been reared on meridic diet since 1971, and a feral population held under controlled conditions for only a few successive generations. However, cumulative larval numbers from SIMRU’s laboratory colony of *H*. *zea* on non-*Bt* cotton fruiting structures were not significantly different from those observed for the feral population collected from Pickens, AR. Although there are likely inherent genetic and phenotypic differences between the two colonies tested, *H*. *zea* larval survival and damage to dual- and multi-gene cotton fruiting structures observed during the study can be attributed mainly to differing levels of susceptibility to *Bt* toxins.

Reports of decreased measurements of heliothine susceptibility to *Bt* toxins in the laboratory have yielded an array of responses regarding insect survival and damage to transgenic plants. Subsequent to an early study conducted in the private sector [18], Luttrell and collaborators published an extensive array of papers that demonstrated the variability in response of *H*. *zea* to *Bt* toxins observed across the landscape [48–51], one of which also predated the release of *Bt* crops in the U.S. [19]. These *H*. *zea* collections were obtained from *Bt* and non-*Bt* crop hosts and wild hosts, and the variability of responses measured were extremely large relative to those reported for other lepidopteran pests of cotton in the U.S. Luttrell et al. [19] also reported that *H*. *zea* possessed “the genetic capacity to develop resistance to endotoxin proteins” prior to the release of *Bt* cotton in the U.S. High densities of *H*. *zea* infesting *Bt* cotton in Alabama [52], Mississippi [53], and North Carolina [54] were reported during the first year of its release in the U.S., with boll damage estimates reaching 24% in some cases. However, these densities and damage levels were not observed the following year, which may be an indirect indicator that fitness costs associated with survival on *Bt* cotton and inherent genetic variability contributed to low heritability of resistance in *H*. *zea*.

Data from this study support findings of field-evolved resistance reported by Reisig et al. [22] that decreased measurements of laboratory susceptibility in *H*. *zea* to Cry1Ac resulted in increased damage to fruiting structures on dual- and multi-gene *Bt* cottons. This study provides evidence that a decreased measurement of susceptibility in *H*. *zea* to Cry1Ac in the laboratory resulted in higher levels of insect survival and plant damage on Bollgard II and Bollgard III cottons relative to a laboratory susceptible colony. These findings were unexpected for Bollgard III, which expresses Vip3A in addition to the Cry1Ac and Cry2Ab2 proteins found in Bollgard II. Vip3A, which is expressed in third generation *Bt* cotton varieties, has been reported as an effective control for *H*. *zea* [55–57]. This vegetative insecticidal protein is produced during different life cycle stages of *Bt* than those crystalline in nature, which may guard against higher population levels of *H*. *zea* and reduce the need for supplemental control with conventional insecticides. This study was intended to simulate a “high-pressure” *H*. *zea* event in the field. The damage inflicted to fruiting structures on Bollgard III cotton by a feral *H*. *zea* colony with decreased measurements of susceptibility to Cry1Ac suggest that the addition of Vip3A to third generation *Bt* cottons may not provide sufficient control in certain situations. Supplemental control of *H*. *zea* in Bollgard III cottons may be required in high-pressure situations where insects with reduced susceptibility to *Bt* toxins are suspected.

Mississippi cotton growers alone spent nearly $15 million for supplemental control of *H*. *zea* in cotton in 2017 [58], which was up from $2.7 and $7.3 million in 2015 [59] and 2016 [60], respectively. If trends for declining efficacy of dual-gene transgenic cottons [61] and increasing supplementary control costs of *H*. *zea* in *Bt* cotton continue, multi-gene cotton varieties will likely be rapidly adopted in the southern U.S. Although this experiment was designed to mimic a “high-pressure” *H*. *zea* situation in cotton, the differences in survival of the tolerant colony relative to the laboratory susceptible colony in Bollgard III was alarming. Therefore, we must exercise prudence regarding resistance management strategies in cotton for Vip3A to preserve its effectiveness against *H*. *zea* that have reduced susceptibility to *Bt* toxins. Additional factors such as selection for resistance, the resultant fitness costs (e.g. stunting, delayed development, and molt inhibition) associated with survival on *Bt* crops, and the dilution of resistance genes due to migration may contribute to variability associated with the survival of *H*. *zea* on dual- and multi-gene *Bt* cottons. These factors combined with a presumed “high dose” of Vip3A expressed in third generation cottons may help delay field-evolved resistance in *H*. *zea* and prolong effectiveness of other *Bt* toxins. Ultimately, this study demonstrated that different responses to Bt toxins between two populations of *H*. *zea* as measured via laboratory bioassays corresponded to differences observed in plant-based studies on Bt cotton.
